# Resting State Dynamic Reconfiguration of Spatial Attention Cortical Networks and Visuospatial Functioning in Non-Verbal Learning Disability (NVLD): A HD-EEG Investigation

**DOI:** 10.3390/brainsci13050731

**Published:** 2023-04-27

**Authors:** Ambra Coccaro, Maria Grazia Di Bono, Antonio Maffei, Camilla Orefice, Rachele Lievore, Irene Mammarella, Mario Liotti

**Affiliations:** 1 Department of Developmental and Social Psychology, University of Padova, Via Venezia 8, 35131 Padova, Italy; 2 Padova Neuroscience Center, University of Padova, Via Orus 2/B, 35129 Padova, Italy; 3 Department of Psychology, Simon Fraser University, Burnaby, BC V5A1S6, Canada

**Keywords:** nonverbal learning disability, resting-state electroencephalography, visuospatial abilities, gamma band, delta band, right hemisphere

## Abstract

Nonverbal learning disability (NVLD) is a neurodevelopmental disorder characterized by deficits in visuospatial processing but spared verbal competencies. Neurocognitive markers may provide confirmatory evidence for characterizing NVLD as a separate neurodevelopmental disorder. Visuospatial performance and high-density electroencephalography (EEG) were measured in 16 NLVD and in 16 typically developing (TD) children. Cortical source modeling was applied to assess resting-state functional connectivity (rs-FC) in spatial attention networks (dorsal (DAN) and ventral attention networks (VAN)) implicated in visuospatial abilities. A machine-learning approach was applied to investigate whether group membership could be predicted from rs-FC maps and if these connectivity patterns were predictive of visuospatial performance. Graph theoretical measures were applied to nodes inside each network. EEG rs-FC maps in the gamma and beta band differentiated children with and without NVLD, with increased but more diffuse and less efficient functional connections bilaterally in the NVLD group. While rs-FC of the left DAN in the gamma range predicted visuospatial scores for TD children, in the NVLD group rs-FC of the right DAN in the delta range predicted impaired visuospatial performance, confirming that NVLD is a disorder with a predominant dysfunction in right hemisphere connectivity patterns.

## 1. Introduction

Nonverbal learning disorder (NVLD) is a neurodevelopmental disorder with a neuropsychological profile characterized by visuospatial processing deficits, within a profile of intact verbal abilities [1,2,3,4]. Individuals with NVLD show major problems with visuospatial working memory (VSWM, i.e., [5,6,7]), visuoconstructive and spatial organizational skills [8,9,10,11], comprehension of spatial descriptions [12,13], and nonverbal problem-solving abilities [14]. Such neuropsychological deficits may be associated with learning difficulties in the areas of mathematics, geometry, and drawing [7,15,16,17]. Finally, studies have also reported difficulties in social interaction abilities for individuals with NVLD [18,19] albeit less pronounced relative to autism spectrum disorder (ASD). Although in recent years researchers have collected many data supporting the main characteristics of NVLD, despite its growing recognition, NVLD is not yet identified in the current classification systems as a distinct developmental disorder (DSM-5, APA, 2013; ICD-11; World Health Organization [WHO], 2018). Since neurobiological markers of brain structure and function may contribute important convergent evidence that NVLD is indeed a distinct disorder, the present study employed behavioral and brain electroencephalographic (EEG) measures in an attempt to discriminate between children with NVLD and with typical development (TD), and to predict performance in the visuospatial domain from connectivity patterns of brain activity. Only a few studies on NVLD using brain-based techniques have been published in the literature, and their findings are summarized below.

### 1.1. Neural Correlates of NVLD

Based on the neuropsychological profile and the presence of mild left-sided motor and sensory signs [20,21], Rourke [1] postulated that the neurological basis of NVLD is a “white matter” syndrome, with a predominant dysfunction in right hemisphere connectivity patterns, a hypothesis based on the available evidence linking the right hemisphere with specialized visuospatial processing [22,23,24,25].

An electroencephalography (EEG) study [26] tested Rourke’s right hemisphere hypothesis employing EEG, comparing two groups of children–one with NVLD and the other with verbal learning disorder (dyslexia). They computed EEG coherence, a frequency-specific measure that reflects functional interregional coupling, mainly depending on structural connections [27,28]. Consistent with Rourke’s hypothesis, in the NVLD group, they found, in the resting state, a relative decrease in coherence in the gamma band between distant locations restricted to the right hemisphere (long-distance hypoconnectivity), interpreted as reflecting defective neuronal interactions between distant cortical regions in the right hemisphere [26]. Albeit promising, the study had some limitations. First, there was no comparison group of typically developing (TD) children. Second, no behavioral performance data in the visuospatial domain were reported. Third, EEG functional connectivity was calculated only at the scalp level from a sparse sensor array (19 electrodes).

Advances in imaging techniques based on magnetic resonance imaging (MRI) have addressed brain correlates of visuospatial and social deficits in NVLD. Concerning the former, an anatomical MRI study measured the volume of the splenium of the corpus callosum, connecting temporal, posterior parietal, and occipital cortices across the two hemispheres. Compared to TD children and other clinical groups (attention deficit–hyperactivity disorder and autism spectrum disorder), the NVLD group showed smaller splenial volumes, which were associated with lower performance IQ but not verbal IQ scores, suggesting that the visuospatial deficits may derive from the inability to integrate visuoperceptual and visuospatial information across the hemispheres [29]. A second MRI study compared resting-state functional connectivity (rs-FC) among children with NVLD and reading disorder (RD), as well as TD children [30]. They analyzed a broad spatial network, including nodes in the dorsal attention network (DAN) involved in VSWM and spatial attention (see below), as well as other cortical areas involved in topographical memory (retrosplenial cortex, parahippocampal gyrus, and others). Across all groups, global network efficiency was associated with performance IQ. Within the spatial network, reduced rs-FC in NVLD relative to the other two groups combined was found between the left posterior cingulate cortex (PCC) and the right retrolimbic area (RA), which correlated with differences between groups in performance IQ [30]. Since the splenium contains fibers directly connecting left and right retrosplenial cortices [31]—which include both PCC and RA—these results could still be accounted for by a white-matter abnormality centered in the splenium, as reported in the previous study [29]. Concerning the neural substrates of social abilities in NVLD, a structural MRI study found smaller volume of the anterior cingulate cortex (ACC) in NVLD relative to typically developing (TD) children [32]. A second study reported reduced rs-FC between ACC and the anterior insula (hubs of the salience network) in NVLD relative to TD children [33].

In recent years, developments of the EEG technique, including high-density sensor arrays and EEG cortical source modeling, allow the study of functional connectivity with much greater detail and spatial resolution than before. ‘Dynamic network neuroscience’ aims to investigate the interconnected nature of neurophysiological phenomena underlying human cognition in health and disease [34,35]. A flexible dynamic reconfiguration of the modular organization of cortical networks has been related to learning proficiency in healthy individuals [34], to memory and executive functions performances [36], and to social cognitive abilities [37]. This approach has recently been successfully employed in network disorders such as temporal lobe epilepsy [38].

### 1.2. Visuospatial Working Memory and EEG Oscillations

In EEG studies with healthy volunteers performing visuospatial processing tasks, findings were reported associating oscillatory patterns to spatial short-term memory, in particular, mental rotation, especially in the gamma band (30–80 Hz) [39,40] but also in the beta band (13–29 Hz) [41]. Of relevance was a review by Tallon-Baudry [42], concluding that, in working memory tasks, gamma and beta bands address different processing stages. The gamma frequency would be prevalent during the presentation of the item to be held in memory, while the beta band would be prominent during the maintenance period. In fact, Von Stein and Sarnthein [43] proposed that the bottom-up or perceptually driven processes are mediated by local gamma frequency, whereas top-down processes would involve long-distant oscillations in the beta, alpha (8–12 Hz), and theta (4–7 Hz) bands. The gamma frequency band has also been linked to perceptual binding, that is, the process whereby the sensory stimuli are combined together in order to create a meaningful and unitary percept.

Importantly, Basso Garcia et al. [44] assessed visual short-term memory for shapes and colors and the binding of shapes and colors by comparing a group of children at risk of NVLD with a control group. They found that the groups did not differ in retention of either shapes or colors, but children at risk of NVLD were poorer than children in the control group in memory for shape-color bindings, exhibiting a binding deficit.

### 1.3. Spatial Attention Networks

The investigation of brain network dynamics through high-density EEG and cortical source modeling may help to elucidate the neural mechanisms underlying cognitive impairment in NVLD. Particularly relevant would be to address potential abnormalities in the dorsal and ventral attention networks (DAN and VAN), two anatomically and functionally distinct cortical systems previously identified by fMRI studies of active attention processing, mainly involved in top-down and bottom-up attention processes, respectively [45].

The DAN supports endogenous attention and comprises the frontal eye fields (FEF) and the intraparietal sulcus (IPS). These core regions have retinotopically organized maps of contralateral space [46], which make them particularly suitable for VSWM processes. Activity in these frontal and parietal areas creates maps of prioritized space that rank the importance of locations in the visual field in accordance with their attentional priority [47]. The priority maps are then used in order to select between competing representations of actions in the motor system or between competing representations of objects in the visual system. In addition, there is a substantial body of research describing the involvement of DAN in two main contexts: the representation of spatial information [47,48], and working memory and sustained attention tasks [49,50].

The VAN supports exogenous attentional processes recruiting areas of the ventral prefrontal cortex (VPFC) and the temporo-parietal junction (TPJ). This network has been found to be lateralized to the right [51], but neuroimaging studies have also highlighted a role of the left hemisphere for the TPJ in attentional processes [52], or a bilateral involvement of the same region [53].

### 1.4. Aims

Based upon these premises, the first aim of the present project was to attempt to discriminate between the NVLD and TD groups employing maps of EEG resting-state functional connectivity (rs-FC) in the DAN and VAN of the left and right hemispheres by applying a machine-learning approach, i.e., a support vector regression (SVR) model. The second aim was to determine whether such rs-FC measures would be predictive of performance in the visuospatial domain. Our final aim was to apply graph theoretical analysis to ascertain whether network topology properties (degree, strength, clustering coefficient, and local efficiency) would be effective measures for discriminating between NVLD and TD groups.

### 1.5. Hypotheses

We hypothesized that the DAN and VAN would show differential resting-state connectivity maps between the two groups and that this information would solely be able to distinguish between children with and without NVLD. Given their relevance for visuospatial and working memory processes, we predicted that these changes mostly affect gamma and beta frequency bands. We further hypothesized that visuospatial performance in NVLD would more robustly depend on connectivity patterns within the DAN—particularly in the right hemisphere, given its role in active visuospatial processing. Finally, we predicted that, by examining each cortical node falling in the two neural networks in the two groups, the NVLD cohort would show differential connectivity patterns and networks’ topology from TD children.

## 2. Materials and Methods

### 2.1. Participants

A total of 32 participants (26 males and 6 females), aged 8 to 16 years old, were selected to take part in the present study. The experimental group included participants diagnosed with NVLD (*n* = 16, 2 left-handed) and participants without any diagnosis (not diagnosed), and for whom a typical development was assumed (TD, *n* = 16; 3 left-handed). Only children who achieved a standard score of 80 or above on the full-scale IQ on the Wechsler Intelligence Scale (WISC-IV; [54]) were included in the sample. All participants were native Italian speakers and had normal or corrected-to-normal vision and hearing. None of them had a history of neurological and/or psychiatric disorders, as reported by an anamnestic interview conducted with parents. NVLD and TD groups were not statistically different regarding chronological age [*F* (1, 30) = 0.049, *p* = 0.827, *η^2^_p_* = 0.002], gender distribution [*χ2* (1) = 0.183, *p* = 0.669], or verbal abilities [*F* (1, 30) = 0.732, *p* = 0.399, *η^2^_p_* = 0.024], as measured using the Vocabulary subtest from the WISC-IV (Wechsler, 2003).

Children with NVLD had previously received an independent clinical diagnosis by private psychologists or child psychiatrists at specialized clinical centers, following recommendations from the literature [55], while children in the TD group were recruited via local schools or community contacts. The Developmental Test of Visual-Motor Integration (VMI; [56]) was used as a screening measure to assess visuospatial processing: the scores of the two groups were significantly different [*F* (1, 30) = 21.550, *p* < 0.001, *η^2^_p_* = 0.519], highlighting the presence of significant impairments only in the NVLD group. Moreover, aiming to perform a differential diagnosis between NVLD and autism spectrum disorder (ASD) without intellectual disability, the Autism Diagnostic Interview-Revised (ADI-R; [57]) was administered to the participants’ parents. All participants, from both the NVLD and the TD groups, scored below the clinical cut-offs in all the assessed areas (i.e., Reciprocal Social Interactions, Language/Communication, and Repetitive Behaviors/Interests). Descriptive statistics concerning inclusion and screening measures are provided in Table 1.

All participants’ parents or legal guardians gave written informed consent before the experiment, and the participants’ agreement to take part in the study was acquired. All experimental procedures were approved by the Ethics Committee of the School of Psychology at the University of Padua (protocol no 3921) and were conducted according to the principles expressed in the Declaration of Helsinki.

### 2.2. Visuospatial Performance

The Rey–Osterrieth complex figure test (ROCFT; [58]) assesses visuoconstructive abilities and visuospatial memory. Participants are asked to copy a complex geometrical figure as accurately as possible. After 3 min, they are requested to reproduce it from memory. Accuracy is determined by scoring each element based on its presence, accurate reproduction, positioning, and respect for proportions [58]. In the present study, individual scores for the copy and recall portions were averaged together for the behavioral prediction from EEG connectivity data (see below).

### 2.3. EEG Resting-State Recording

For each participant, the rs HD-EEG activity was recorded before the active tasks (not reported here) in a 4-min eyes-closed session. We used a geodesic high-density EEG system (EGI^®^ Net Amp GES-400) (Magstim EGI, Withland, UK) with a pre-cabled 256 channels, through NetStation (v5.4) EEG Software. The elastomer structure of the EEG net is formed by polyvinyl alcohol sponges that are housed within the HydroCel Hydrating Skin interface chamber. The sampling rate of the recording was set to 500 Hz, with an automatic alignment of real-time EEG.

### 2.4. EEG Preprocessing

The preprocessing was performed in MATLAB (v2019b) using functions from the EEGLab (v.2020.048) and ERPLab (v.2021.8.30) Toolbox. Continuous data were downsampled to 256 Hz, a passband filter (0.01–80 Hz) was applied, and the signal was re-referenced to the average of all channels. Next, the clean_artifacts routine in EEGLab was used with default parameters to detect bad channels and exclude them from further processing. If a channel was rejected, its value was replaced by means of the interpolation using surrounding channels. Another cleaning procedure was applied with an ERPLab function, continuousartdet, to reject segments of data with a peak-to-peak amplitude exceeding ±100 μV using a moving-window procedure (window size = 200 ms, step size = 20 ms). Finally, independent component analysis (ICA) was performed, and artifact components were marked with ICLabel and manually discarded.

### 2.5. EEG Source Modeling and Connectivity Analysis

The processing phase was carried out with Brainstorm and Matlab (MathWorks, Inc.). In order to model the source activity, a forward model was calculated with the BEM, a three-layer boundary element method, and the source was estimated with the weighted minimum norm estimation (wMNE) method. This inverse solution was then downsampled to 148 cortical parcels defined by the Destrieux Atlas [59]. The connectivity matrices were calculated with magnitude squared coherence (MSC), which describes the linear relationship (covariance) between two signals in the frequency domain (Delta: 2–4 Hz, Theta: 5–7 Hz, Alpha: 8–12 Hz, Beta: 13–29 Hz, Gamma: 30–59 Hz), and it is calculated as follows:

(1) $$\begin{array}{c} |\;\;C_{xy}(ƒ)\;\;{|}^{\;\;2}\;={\left(\frac{\left|S_{xy}\left(ƒ\right)\;\right|}{\sqrt{S_{xx\;}\left(ƒ\right)S_{yy\;}\left(ƒ\right)}}\right)}^{2}\\ S_{xy}\left(ƒ\right)\;:\;\mathrm{Cross-spectrum}\\ S_{xx\;}\left(ƒ\right)\;and\;S_{yy\;}\left(ƒ\right)\;:\;\mathrm{Auto-spectra}\;\mathrm{or}\;\mathrm{power}\;\mathrm{spectral}\;\mathrm{density} \end{array}$$

Thus, the MSC (C) between two signals (*x* and *y*) is estimated by the square of the coherence value between x and y divided by the square root of the coherence of *x*, with *x* multiplied by the cohere of *y* with *y*.

### 2.6. Discrimination between NVLD and TD Groups: A Machine-Learning Approach

After EEG signal preprocessing, source-reconstructed cortical activity and whole-brain resting-state functional connectivity (rs-FC) were computed. Subsequently, phase coherence values were extracted from the parceled cortex (Destrieux atlas, 148 ROIs; [59]) to estimate individual rs-FC in the DAN and the VAN. These cortical networks were distinguished by hemisphere, given the recognized role of the right hemisphere for visuospatial processing [23,24,25]. A machine-learning approach (i.e., support vector machine) was applied in order to investigate whether group membership could be predicted from rs-FC maps in each hemisphere and frequency band (Delta: 2–4 Hz, Theta: 5–7 Hz, Alpha: 8–12 Hz, Beta: 13–29 Hz, Gamma: 30–59 Hz). The objective of this first analysis was to investigate whether (i) there was such information, within the coherence maps of the selected networks of interest, able to discriminate between the NVLD and TD groups and (ii) the hemisphere played a crucial role in differentiating the two groups. In order to test these hypotheses, we used a machine-learning approach based on the SVM classifier. The Matlab functions svmtrain and svmclassify, respectively, were used in order to train a linear SVM model (with default parameters) for discriminating between the clinical and the control groups, starting from the functional connectivity matrices. The Matlab function cvpartition was employed, at each run, for implementing the leave one subject out cross-validation scheme. The prediction accuracy was computed at the end of the cross-validation loop on the corrected predicted classes (one per each test subject, at each run).

### 2.7. Behavioral Predictions from Functional Connectivity Matrices

In order to understand the characteristics driving the successful classification of FC maps of NVLD with respect to TD children, we applied a series of linear regression models. The goal of this approach was to link the functional connectivity information, which could discriminate the NVLD group from the control one, to the individual visuospatial performance in the ROCFT. We adopted an approach similar to that used by Duma et al. [60]—specifically, an SVR (support vector regression) model for each group, network of interest (considered separately in the left and right hemispheres), and frequency band. For testing the generalization ability of the regression model, we adopted a leave-one-subject-out cross-validation scheme, implemented across subjects within each group. As suggested by Yadav et al. [61], leave-one-out cross validation is preferred for datasets with a sample number less than 100. By contrast, for very large datasets, using this cross-validation scheme could increase the overfitting probability, and therefore, other schemes, like folds, can be used [61,62]. The prediction accuracy was expressed in terms of a correlation coefficient (see [60,63] for a similar procedure applied, respectively, to EEG and fMRI data). For more details about the analysis, see Section 2.9 in the work of Duma et al. [60]. Only positive correlations were reported as an index of a good-quality fitting. Note that negative correlations are an index of a very bad fitting and therefore were not considered. Using Bayesian correlation allowed us to get a ratio between the null hypothesis and the alternative hypothesis and provided a measure of the strength of evidence of one hypothesis over the other, which is highly valuable in clinical research. In the frequentist approach, instead, the *p*-value is not informative for the alternate hypothesis; it only computes the likelihood of the null hypothesis and allows researchers to discard it. Moreover, the use of the Bayes factor robustness check allowed us to test the robustness of the obtained results by varying the beta-prior width. Bayes factor (BF) correlations were performed using the software JASP (https://jasp-stats.org/ accessed on 6 November 2014). Note that the reported BF values correspond to the assumption of a Cauchy prior width equal to 0.5, but a BF robustness check was considered in order to estimate the robustness of the results as a function of the selected prior width. Considering the scale of interpretation of the BF (Jeffreys, 1998), we adopted a conservative approach, reporting only the correlations with BFs ≥ 3, which is considered moderate (3 < = BF < 10) to strong (10 < = BF < 30), very strong (30 < = BF < 100), or extreme (BF > = 100) evidence toward the alternative hypothesis.

### 2.8. Discrimination between NVLD and TD: A Graph Theory Approach

#### 2.8.1. Graph Construction

We used a single-subject-connectivity-matrix approach, as suggested by Langer et al. [64]. Thus, for each network of interest, frequency band, and subject, we constructed a graph (N × N adjacency matrix, where N represents the number of ROIs included in the network), and then we extracted the graph measures (i.e., 3 global measures and 3 local measures) by using the Brain Connectivity Toolbox (BCT). Note that graph connections were not binarized, in order to avoid a loss of information. Moreover, we maintained all the weighted connections in graph construction under the threshold of 10% of the top-ranked connections. For reasons related to the numerosity of nodes in our selected networks (i.e., twelve nodes in both hemispheres), we preferred a more conservative approach considering only the most informative coherence values in the graph. Indeed, in this way, we preserved the network topology and retained the maximum information within the graphs, avoiding spurious connections.

#### 2.8.2. Graph Measures

All the measures were computed on the weighted graphs normalized by using the function weight conversion (with the parameter option “normalize”), contained in the BCT for normalizing the graph connectivity. This function scales all weight connections to the range [0, 1] by dividing the connection values by the maximal weight and should be performed prior to computing the network parameters (e.g., clustering coefficient). Indeed, since network measures strictly depend on the mean of the weighted connections, weighted graphs need to be normalized in order to perform statistical analysis on the extracted measures. We extracted graph measures both at a global and a local (i.e., nodal) level. For global measures, we tested the hypothesis of a difference between the NVLD and the TD control group, considering potential hemispheric asymmetries. Thus, we extracted the global measures from the graphs computed separately for each hemisphere (e.g., left DAN and right DAN graphs). At the nodal level, we extracted the graph measures from the complete graphs, considering both the hemispheres in a single graph (i.e., bilateral DAN and VAN).

#### 2.8.3. Global Measures

In order to characterize both segregation and integration properties of each functional network at rest, we extracted three global measures (i.e., one value per subject for each graph of interest, in each hemisphere—L DAN, R DAN, L VAN, R VAN): (i) global efficiency, which can index both segregation and integration functional properties; (ii) assortativity, which can index the presence of hierarchy in structuring the information flow; and (iii) modularity, which can index segregation (i.e., specificity) in information elaboration.

#### 2.8.4. Nodal Measures

At a nodal level, we extracted two local measures (i.e., one value per subject for each node within the graph of interest—DAN and VAN) from each considered brain network modeled as a graph: (i) *degree, which is the number of connections incident on the node, surviving after normalization of the adjacency matrix, and could be interpreted as an index of integration*; (ii) *strength, which is computed for each node of the graph as the sum of the weights on its connections and could be interpreted as an index of integration and synchronization of brain activity*; and (iii) *clustering coefficient, which quantifies*, for each node, how close its neighbors are to becoming a complete graph; it reflects the prevalence of clustered connectivity around individual nodes and roughly corresponds to an index of segregation and specialization.

#### 2.8.5. Statistical Analyses between NVLD and TD

We performed Bayesian independent sample *t*-tests using the software JASP (https://jasp-stats.org/), reporting the Bayes factor (BF) in favor of the alternative Hypothesis H1 (NVLD ≠ TD). As described for the correlation analysis, we used a Bayesian independent *t*-test that provides a measure of the strength of evidence of the alternative hypothesis over the null hypothesis, by considering the previously mentioned parameters.

## 3. Results

### 3.1. Visuospatial Performance Measures

As expected, the NVLD group and the TD control group significantly differed both in the copy and the memory parts of the Rey–Osterrieth complex figure test, with the worst performance in the NVLD group. For consistency with the EEG connectivity approach, results in Table 2 employed Bayesian independent sample *t*-tests. Note that the results stayed the same when employing traditional F-tests for the ROCFT copy [*F* (1, 30) = 29.67, *p* < 0.001, *η^2^_p_* = 0.497] and the ROCFT recall [*F* (1, 30) = 22.19, *p* < 0.001, *η^2^_p_* = 0.425].

### 3.2. Rs-Connectivity Differences in the DAN and in the VAN

The support vector machine model highlighted the fact that our selected networks contained information able to discriminate between the NVLD and TD groups. Specifically, classification results showed that the DAN in both hemispheres (left DAN: accuracy of 62.5% in the beta band, 62.5% in the delta band, and 84.38% in the gamma band; right DAN: accuracy of 56.29% in the alpha band and 59.38 in the delta band), and the VAN in both hemispheres (left VAN: accuracy of 65.63% for the beta band; right VAN: accuracy of 59.38% in the alpha, beta, and gamma bands, and 65.63% in the theta band) contained functional connectivity information able to discriminate (above the chance level of 50%) between the two groups. We focused our analysis on graph-theory measures on those frequency bands that were most informative, on average, across networks and hemispheres, in discriminating between the two groups: i.e., beta (M = 57.03, SE = 2.81) and gamma (M = 56.25, SE = 5.34) bands. For the remaining frequency bands, the mean accuracy across hemispheres and networks did not exceed the chance level of 50% (alpha: M = 49.22, SE = 10.33; delta: M = 50, SE = 15.52; theta: M = 42.19, SE = 20.65). In order to better understand and characterize these findings, we applied successive analyses based on graph theory and regression models, focusing on the beta and gamma frequency bands.

### 3.3. Behavior Prediction from Functional Connectivity Matrices

**Figure 1:**
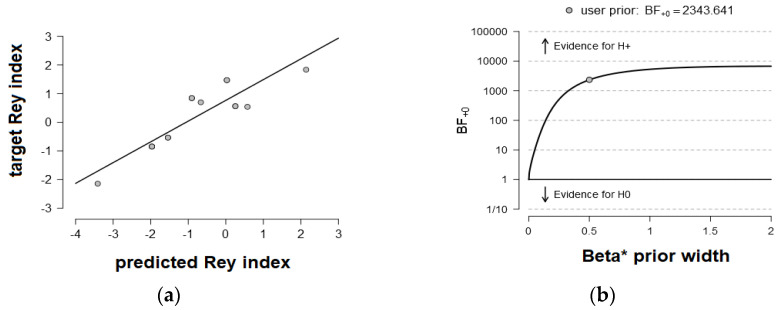
Prediction of the visuospatial performance from the connectivity maps in TD children. (**a**): Prediction of visuospatial individual performance (as indexed by the Rey index) from rs-functional connectivity within the left dorsal attention network (DAN; R = 0.89, BF_10_ = 2343.64) in TD children in the gamma frequency band. (**b**): BF robustness check showing that the evidence was preserved as extreme (BFs_10_ > 1000) by varying the Cauchy prior width.

**Figure 2:**
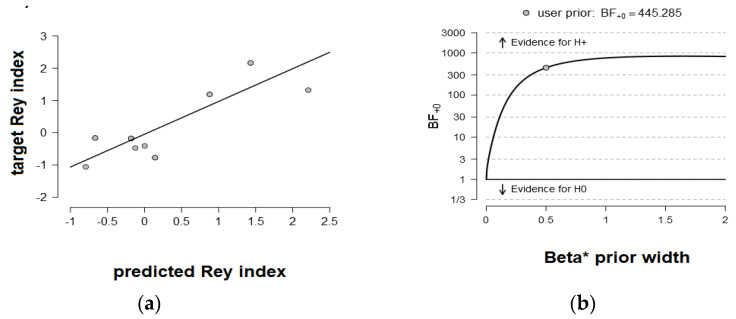
Prediction of the visuospatial performance from the connectivity maps in NVLD children. (**a**): Prediction of visuospatial individual performance (as indexed by the Rey index) from rs-functional connectivity within the right dorsal attention network (DAN; R = 0.84, BF_10_ = 445.28) in NVLD children in the delta frequency band. (**b**): BF robustness check showing that the evidence was preserved as extreme (BF_10_ > 100) by varying the Cauchy prior width.

Results presented in Table 3 showed a differential pattern of predictions in NVLD and TD children. In the TD group, rs-functional connectivity in the left DAN in the gamma band (R = 0.89, BF_10_ = 2343.64, extreme evidence) and in the left VAN in the delta band (R = 0.55, BF_10_ = 5.65, moderate evidence) was predictive of visuospatial abilities in the ROFC. In sharp contrast, in the NVLD group, rs-functional connectivity in the right DAN in the delta band was predictive of the visuospatial performance level (R = 0.84, BF_10_ = 445.29, extreme evidence). Figure 1b and Figure 2b show the BF robustness checks for the predictions where the evidence was preserved as extreme (BFs_10_ > 100) by varying the Cauchy prior width.

### 3.4. Discrimination between NVLD and TD: A Graph Theory Approach

#### 3.4.1. Global Measures

**Figure 3:**
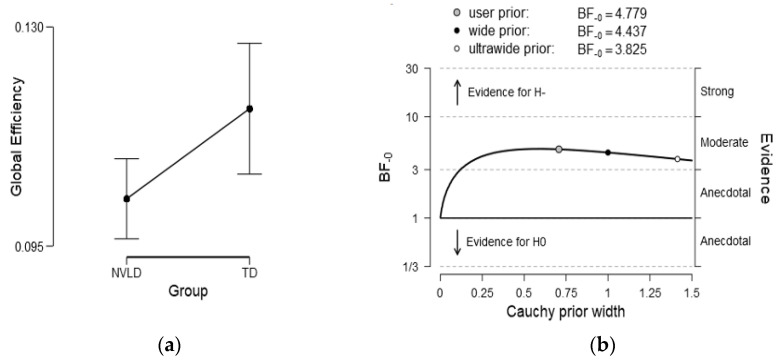
(**a**): Mean global efficiency for the NVLD children, which was lower than that of the controls (BF_10_ = 4.78) in the left VAN (in the beta frequency band). (**b**): BF robustness check showing that the evidence was preserved as moderate (3 < BFs_10ß_ < 10) by varying the Cauchy prior width.

**Figure 4:**
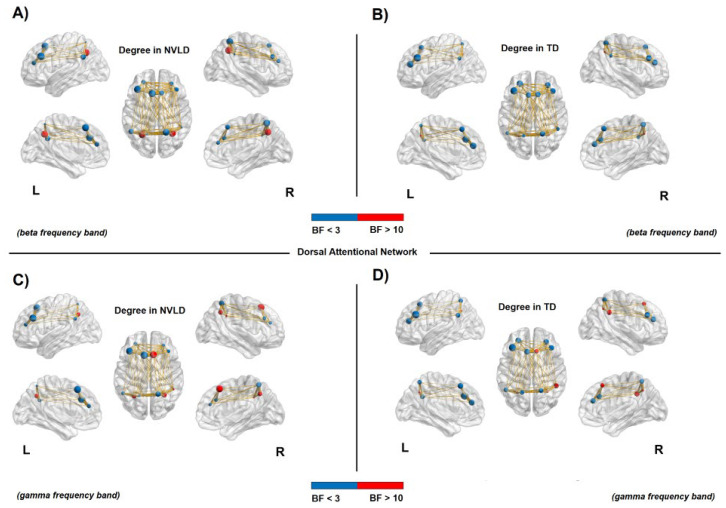
Connectivity degree in the dorsal attention network. Node size represents the value of degree, whereas node color relates to the BF value: red for a BF > 10 and blue for a BF < 3. The red nodes report evidence for a significant difference between NVLD and TD children. (**A**,**B**): Increased connectivity degree in the bilateral intraparietal sulcus (IPS) for the NVLD group compared to TD controls, in the beta frequency band. (**C**,**D**): Increased connectivity degree for NVLD children compared to TD controls, in bilateral IPS and areas of the frontal eye field (FEF) in the right hemisphere in the gamma frequency band (see Table A1 for details on statistics).

At a global level, we tested the directional hypothesis that the global efficiency of the NVLD group is lower than that of the TD control group. Bayesian independent sample *t*-tests showed that in the left VAN (in the beta band), there was moderate evidence of a lower mean global efficiency for the NVLD relative to the control group children (BF_10_ = 4.78; NVLD: M = 0.10, SE = 0.003; TD: M = 0.12, SE = 0.005), as displayed in Figure 3. No effect on global efficiency emerged in the gamma band (BF_10_ = 1.45), nor did other effects emerge when considering assortativity (BF_10_= 0.18) or modularity (BF_10_= 0.2) across networks and hemispheres. As shown in Figure 4B, the BF robustness check showed that the evidence was preserved as moderate (3 < BF_10_ < 10) by varying the Cauchy prior width.

#### 3.4.2. Nodal Measures: Dorsal Attention Network

**Figure 5:**
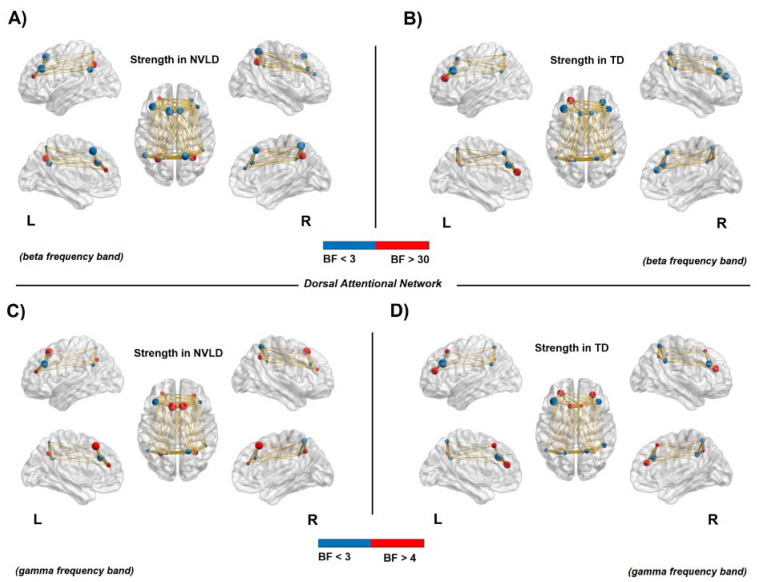
Connectivity strength in the dorsal attention network. Node size represents the strength value, whereas the color relates to the BF value: red for a BF > 30 in panels (**A**) and (**B**), and BF > 4 in panels (**C**) and (**D**); blue for a BF < 3. The red nodes report evidence for a significant difference between NVLD and TD children. (**A**,**B**): Increased connectivity strength in the bilateral frontal eye field (FEF) areas within the dorsal attention network for NVLD children compared to TD controls in the beta frequency band. (**C**,**D**): Increased connectivity strength for NVLD children compared to TD controls in bilateral IPS and areas of the FEF in the right hemisphere in the gamma frequency band (see Table A2 for details on statistics).

Results from Bayesian independent sample *t*-tests showed an *increased* connectivity in the bilateral DAN regions (i.e., frontal eye fields—FEF and intraparietal Sulcus—IPS, bilaterally) for NVLD children compared to TD controls, as shown by the degree and strength measures in both beta and gamma frequency bands (see Table A1 and Table A2 in Appendix A for statistical information, and Figure 4 and Figure 5).

**Figure 6:**
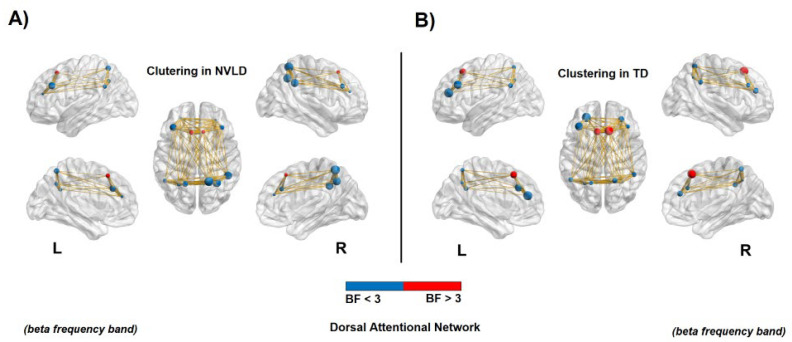
Connectivity clustering in the dorsal attention network. Node size represents the clustering value, whereas node color relates to the BF value: red for a BF > 3 and blue for a BF < 3. The red nodes report evidence for a significant difference between NVLD and TD children. (**A**,**B**): Decreased clustering coefficient for NVLD children compared to TD controls, in bilateral areas of the frontal eye field (FEF) in the beta frequency band (see Table A3 for details on statistics).

Instead, bilateral frontal areas of the DAN showed a decreased local specificity in NVLD children with respect to the TD controls, as measured by a reduced clustering coefficient in the beta band (see Table A3 in Appendix A for statistical information, and Figure 6).

#### 3.4.3. Nodal Measures: Ventral Attention Network

**Figure 7:**
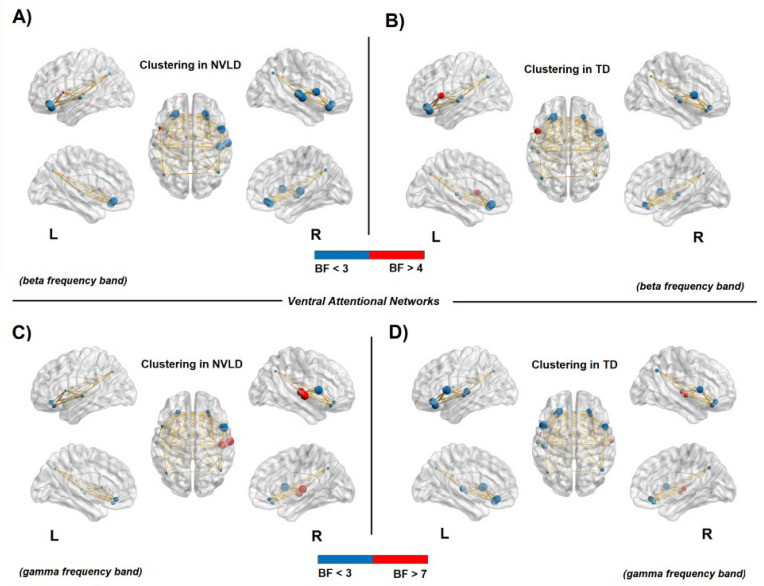
Connectivity clustering in the ventral attention network. The node size represents the clustering value, whereas node color relates to the BF value: red for a BF > 4 in panels (**A**,**B**), BF > 7 in panels (**C**) and (**D;**) and blue for a BF < 3. The red nodes report evidence for a significant difference between NVLD and TD children. (**A**,**B**): Decreased clustering coefficient in the left frontal areas for NVLD children compared to TD controls in the beta frequency band. (**C**,**D**): Increased clustering coefficient for NVLD children compared to TD controls in areas within the right temporoparietal junction (TPJ) in the gamma frequency band (see Table A4 for details on statistics).

Results from Bayesian independent sample *t*-tests showed a decreased clustering coefficient in the left ventral prefrontal cortex (in the beta band) for the NVLD children, with respect to the controls, as an index of reduced local specificity. The opposite pattern of results emerged in the right temporoparietal junction (in the gamma band), where an *increased* clustering coefficient was found for the NVLD group with respect to the TD group (see Table A4 in Appendix A for statistical information, and Figure 7).

## 4. Discussion

Visuospatial performance levels and EEG resting-state functional connectivity in spatial attention networks were investigated in children with nonverbal learning disability (NVLD) and in typically developing (TD) children. A machine-learning approach was employed to test whether the two groups could be discriminated by EEG connectivity patterns. The resting-state connectivity maps were then employed to predict the individual performance in the visuospatial domain. Finally, a graph theoretical approach was employed to determine whether network topology properties (degree, strength, clustering coefficient, and local efficiency) would be effective measures for discriminating between NVLD and TD groups.

At the neural level, rs-FC maps contained information able to differentiate children with and without NVLD. The SVM approach turned out to be useful in selecting the most informative and discriminative frequency bands, namely, the beta and the gamma bands, for which we explored, more in depth, the internal organization of the neural nodes in the DAN and VAN using the graph theory measures. We found an increased connectivity in parietal and frontal areas of the DAN in children with NVLD compared to TD children. Critically, the maps relative to the left DAN in the gamma frequency band were able to predict visuospatial performance for children without NVLD, whereas in the NVLD groups, we found the same prediction on the right DAN in the delta frequency band.

### 4.1. Behavioral Measures

As expected, performance of the Rey–Osterrieth complex figure test was significantly different in children with and without NVLD, as previous research reported [8,11,16,66], reflecting the presence of core visuospatial and visuoconstructive processing deficits among the NVLD children.

### 4.2. EEG Rs-Functional Connectivity: Discrimination between NVLD and TD

The support vector machine model proved that the selected networks of interest (DAN and VAN) contained information able to reliably discriminate between the NVLD and TD groups, based on resting EEG functional connectivity in the gamma and beta bands, which are implicated in visuospatial and working memory processing in healthy participants [39,40,41]. The centrality of gamma band abnormalities confirms the results of a previous resting EEG study comparing a NVLD group to a group with verbal LD, which reported a decrease in gamma band coherence between distant locations in the right hemisphere [26].

### 4.3. EEG Rs-Functional Connectivity: Behavior Prediction

An important and novel finding of this study is that, while for the control TD children, increased resting-state connectivity (rs-FC) in the DAN in the gamma band (in the left hemisphere) strongly predicted visuospatial performance, in the NVLD group, rs-FC in the delta band in the right DAN strongly predicted individual visuospatial performance. The results in the TD children confirmed the role of gamma rhythm in spatial and working memory processes in healthy individuals [39,40]. The different prediction profile in NVLD can be explained by previous resting EEG findings in verbal learning disabilities reporting a preponderance of slow-frequency activity (“slowing”) [67,68,69]. In one such study, more delta power in frontotemporal regions predicted worse educational evaluations in children with verbal learning disorder, interpreted as a sign of underlying cerebral dysfunction in areas involved in reading and writing processes [70].

Importantly, it has been proposed that the slower EEG activity of children with an LD is akin to that of younger, healthy children since slow EEG activity in the delta range is prevalent in early life, later replaced by faster rhythms [71]. This apparent lag in the brain functional development of children with LDs has led to the hypothesis that a delay in the maturation of the brain’s electrical activity impairs children’s ability to achieve their academic milestones [69]. In agreement with the above studies and their conclusions, we interpret the NVLD-specific behavioral prediction with rs-FC in the right DAN in the present study as indicating a suboptimally functioning resting-state network that provides a detrimental ‘starting point’ for task-specific brain activations, pinpointing an inefficient neural resource control due to a delay in neural maturation.

### 4.4. EEG Rs-Functional Connectivity: Discrimination between NVLD and TD with a Graph Theory Approach

In this paper, we also reported novel evidence of reconfiguration of resting-state functional connectivity in spatial attention networks relevant to core symptoms in the visuospatial domain in NVLD. Within the DAN, we found evidence of bilaterally increased functional connectivity at rest for NVLD compared to the TD group, measured through the graph measures of degree and strength. Importantly, however, such increased functional connectivity was associated with reduced local specificity in the frontal nodes of the same network for NVLD children (FEF). The specificity was calculated with the clustering coefficient, which represents the propensity of the network to segregate in order to execute specialized processes. In other words, in the frontal nodes, such connections were more diffuse and less modular: They were not directed within the neural nodes composing the functional network.

Within the VAN, the global efficiency of the left VAN was found to be reduced in the NVLD group. Furthermore, children with NVLD exhibited reduced local specificity (segregation) in the ventral prefrontal (opercular) areas, coupled with an opposite pattern of increased clustering (segregation) in the posterior node of the temporo-parietal junction (TPJ) of the right hemisphere.

### 4.5. Reconfiguration of Rs-Functional Connectivity in NVLD

The present study reports a substantial reconfiguration of resting-state connectivity of the spatial attention networks in NVLD. We propose here that in NVLD, slow rs-EEG connectivity in the delta band, predicting visuospatial performance in the NVLD group, may index immature functional interregional coupling, as a consequence of a primary white-matter abnormality in the right dorsal attention network, specialized for active aspects of visuospatial processing compromised in the disorder, while the bilateral increased gamma connectivity in the DAN, combined with the more diffuse (less modular) gamma connectivity in the frontal nodes, may represent secondary plastic changes in an ineffective attempt to compensate for the primary connectivity dysfunction. Similarly, reconfiguration of functional connections in the VAN in NVLD may constitute an additional compensatory mechanism recruiting more right TPJ, typically activated by exogenous, bottom-up, more automatic spatial orienting of targets [45].

### 4.6. Hemispheric Differences

The behavioral prediction findings in the NVLD group concerning the delta band provide evidence that a primary abnormality at the level of the right dorsal attention network may explain visuoconstructive and visuospatial deficits in NVLD, bringing some support to the centrality of intact right-hemisphere white-matter fibers in this neurodevelopmental disorder [1]. We propose that the primary right DAN abnormality and its effects on visuospatial performance in NVLD may be similar to the effects of lateralized hippocampal lesions on verbal and nonverbal (visual) long-term memory abilities, for example, in patients with temporal lobe epilepsy [72,73]. As proposed for other neurodevelopmental disorders, such abnormalities may take place early in development, likely prenatally, as the result of a combination of genetic and early environmental influences [74]. In contrast, as reported earlier, the bilateral and more diffuse increase in gamma connectivity in the DAN in NVLD, particularly in the frontal nodes, may be the result of later and secondary plastic changes in an ineffective attempt to compensate for the primary connectivity dysfunction, possibly related to a different stage of information processing or a working memory subcomponent (see below).

Another explanation, however, is necessary to interpret the finding that gamma band connectivity at rest in the *left* DAN predicted visuospatial performance in the neurotypical group. A body of evidence in the literature suggests that the verbal/left visuospatial/right dichotomy may be too simplistic. First, based on a meta-analysis of neuroimaging studies, Smith and Jonides concluded that the prefrontal cortex is the main site of working memory but that different regions are specialized for various working memory aspects (storage of spatial, verbal, or visual (object) material, executive processes). While short-term storage of spatial information would be a function of the right hemisphere prefrontal and premotor cortex, short-term storage of visual (object) information (such as memory for non-namable or abstract visual shapes, such as visual matrices) would depend more on left hemisphere prefrontal areas. Furthermore, executive processes required for active manipulation, such as inhibition of irrelevant distractors, may involve the left prefrontal region more [75]. Other neuroimaging studies in healthy volunteers reported increased activity in the DAN bilaterally (both frontal and parietal nodes) in both verbal and visual working memory tasks [76,77,78]. Secondly, an in-vivo structural MRI study in brain-injured patients using the voxel-based lesion symptom mapping approach reported that visuospatial working memory, measured by visuospatial span, was most impaired for lesions in the *left* hemisphere centered on areas of the frontoparietal network, such as the FEF [79]. This confirmed earlier lesion correlation evidence reporting both verbal and visuospatial short-term memory deficits in left hemisphere patients with aphasia [80,81,82]. Based on the evidence mentioned above, our findings of a prediction of functional connectivity in the *left* DAN to visuospatial performance for the TD children are therefore not in contrast to extant literature.

Finally, it is worth cautioning about generalizing conclusions on the hemispheric specialization and the effect of lesions from adult populations to children and adolescent typical and atypical groups.

## 5. Limitations and Future Directions

Because of their relevance to the visuospatial processing symptoms in NVLD, and since we wanted to avoid issues related to significance of multiple comparisons, the present study used a network of interest approach, only exploring resting state functional connectivity in cortical networks clearly associated with spatial attention (i.e., DAN and VAN). However, other cortical networks may be relevant in association with NVLD symptoms. One of them may be the default mode network (DMN), which has been associated with social cognition and whose abnormality may explain the mild deficits of NVLD individuals in social abilities. A previous resting-state fMRI study comparing NVLD, reading disorder (RD), and TD children found reduced connectivity in NLVD only among posterior DMN regions of both hemispheres [30].

It could be argued that the sample size of the clinical group was relatively small. However, NVLD is a rare disorder, and we included participants referred from all over Italy. Futures studies may include patients from cohorts across multiple research institutions and nations. On the other hand, published studies using behavioral [7,9,17] and neural [26,30,33] measures in NVLD and other neurodevelopmental disorders have used comparable sample sizes.

Furthermore, it would be important to test the specificity of the present EEG connectivity abnormalities in NVLD, extending the same approach to other neurodevelopmental disorders and, first, to autism spectrum disorder, which shares some symptoms in the social domain, albeit of different extent/severity. Finally, it would be important to include measures of social ability such as perspective taking or theory of the mind [83,84] in order to assess differences between the two clinical groups.

Lastly, since our study strongly implicates a primary functional connectivity abnormality in the right DAN, it may be important to conduct in NVLD children a MRI diffusion tensor (DTI) tractography study of the superior longitudinal fasciculus in the right hemisphere, particularly the superior longitudinal fasciculus II component connecting the caudal inferior parietal cortex to the dorsolateral prefrontal cortex (dlPFC), providing dlPFC with parietal cortex information regarding perception of visual space [85,86], since this is the primary white-matter tract we suspect to be compromised in NVLD.

## 6. Conclusions

The above limitations notwithstanding, this is the first study to assess resting-state functional connectivity measures in the DAN and VAN using a state-of-the-art graph metrics approach, providing important clues about the topology of functional maps in NVLD and TD groups as a function of the hemisphere. It is also the first to report that such rs-FC measures can discriminate between children with and without NVLD and to discover an association between visuospatial processing and the dorsal attention network in the right hemisphere of NVLD children as a confirmation that the visuospatial deficit is linked to a right hemisphere pathological process, while an opposite left hemisphere association is present in neurotypically developing children.

## Figures and Tables

**Table 1:** Descriptive statistics for the inclusion and screening measures.

Measures	NVLD (*n* = 16) Mean (SD)	TD (*n* = 16) Mean (SD)	Group Differences
Chronological age (months)	157.19 (21.78)	155.44 (23.09)	NS *
Gender (M:F)	12:4	14:2	NS
Vocabulary ^1^	11.56 (2.53)	12.25 (1.98)	NS
VMI ^2^	77.92 (11.96)	101.60 (11.86)	NVLD < TD
ADI-R ^3^: A (Reciprocal Social Interactions)	6.31 (4.99)	2.29 (2.28)	both groups < clinical cut-off
ADI-R ^3^: B (Language/ Communication)	4.88 (3.65)	2.12 (2.62)	both groups < clinical cut-off
ADI-R ^3^: C (Repetitive Behaviors/Interests)	2.64 (2.37)	0.41 (0.62)	both groups < clinical cut-off

^1^ Scaled scores on Vocabulary subtest from the WISC-IV [54]. ^2^ Standard scores in the Developmental Test of Visual-Motor Integration (VMI; [56]). ^3^ Autism Diagnostic Interview-Revised (ADI-R; [57]); higher raw scores reflect more severe impairments in each domain. * *p* > 0.05.

**Table 2:** Descriptive statistics for the copy and recall trials of the Rey–Osterrieth complex figure test.

Measures	NVLD (*n* = 16) Mean (SD)	TD (*n* = 16) Mean (SD)	BF_10_	Group Significance
ROCFT copy trial ^1^	−4.93 (2.69)	−0.88 (1.27)	44,791.26	NVLD < TD
ROCFT recall trial ^1^	−2.52 (1.15)	−0.56 (1.21)	357.90	NVLD < TD

^1^ Z-scores on the Rey–Osterrieth complex figure test [58,65].

**Table 3:** Results of the regression analysis, including the coherence level in the networks and the visuospatial performance in the two groups separately. The Bayes factor is related to the robustness check on the analysis [10 > BF > 3 = moderate evidence, 30 > BF > 10 = strong, 100 > BF > 30 = very strong, BF > 100 = extreme].

ROI	Freq. Bands	Group	BF_10_
R-DAN	delta	NVLD	445.285
L-DAN	gamma	TD	2343.641
L-VAN	delta	TD	5.645

## Data Availability

Data are stored and kept in archived form by the supervisor of the study (M.L.).

## Appendix A

**Table A1:** Bayesian independent samples *t*-test (BF = Bayes factor) and group descriptive (mean, SD = standard deviation, and SE = standard error) of the nonverbal learning disability (NVLD) and the control (TD) groups. The dependent variable is the connectivity *degree* in the *dorsal attention network* in both beta and gamma frequency bands.

Beta Frequency Band	BF₁₀	Group	*n*	Mean	SD	SE
**L S_intrapariet_and_P_trans**	40.70	NVLD	16	1.438	1.031	0.258
		TD	16	0.313	0.602	0.151
**R S_intrapariet_and_P_trans**	10.46	NVLD	16	1.438	0.892	0.223
		TD	16	0.375	1.025	0.256
**Gamma frequncy band**	**BF₁₀**	**Group**	***n***	**Mean**	**SD**	**SE**
**R G_front_sup**	3.521	NVLD	16	1.813	0.834	0.209
		TD	16	1.000	0.966	0.242
**L S_intrapariet_and_P_trans**	218.118	NVLD	16	1.063	0.854	0.213
		TD	16	0.063	0.250	0.063
**R S_intrapariet_and_P_trans**	20.932	NVLD	16	1.250	0.856	0.214
		TD	16	0.250	0.775	0.194
**R S_interm_prim-Jensen**	8.123	NVLD	16	0.500	0.730	0.183
		TD	16	1.250	0.683	0.171

**Table A2:** Bayesian independent samples *t*-test (BF = Bayes factor) and group descriptive (mean, SD = standard deviation, and SE = standard error) of the nonverbal learning disability (NVLD) and the control groups (TD). The dependent variable is the connectivity *strength* in the *dorsal attention network* in both beta and gamma frequency bands.

Beta Frequency band	BF₁₀	Group	*n*	Mean	SD	SE
**L S_intrapariet_and_P_trans**	35.45	NVLD	16	0.951	0.728	0.182
		TD	16	0.178	0.407	0.102
**L S_front_middle**	31.10	NVLD	16	0.684	0.632	0.158
		TD	16	1.399	0.464	0.116
**R S_intrapariet_and_P_trans**	39.96	NVLD	16	0.991	0.715	0.179
		TD	16	0.163	0.512	0.128
**Gamma frequency band**	**BF₁₀**	**Group**	***n***	**Mean**	**SD**	**SE**
**G_front_sup R**	1528.70	NVLD	16	1.362	0.534	0.133
		TD	16	0.448	0.438	0.109
**S_intrapariet_and_P_trans L**	83.332	NVLD	16	0.696	0.660	0.165
		TD	16	0.018	0.071	0.018
**S_front_middle L**	5.103	NVLD	16	0.709	0.612	0.153
		TD	16	1.261	0.517	0.129
**G_front_sup L**	7.685	NVLD	16	1.391	0.696	0.174
		TD	16	0.724	0.567	0.142
**S_intrapariet_and_P_trans R**	12.695	NVLD	16	0.830	0.659	0.165
		TD	16	0.150	0.528	0.132
**G_front_middle R**	4.452	NVLD	16	0.608	0.651	0.163
		TD	16	1.100	0.338	0.084

**Table A3:** Bayesian independent samples *t*-test (BF = Bayes factor) and group descriptive (mean, SD = standard deviation, and SE = standard error) of the nonverbal learning disability (NVLD) and the control groups (TD). The dependent variable is the *clustering coefficient* in the *dorsal attention network* in the beta frequency bands.

Beta Frequency Band	BF₁₀	Group	*n*	Mean	SD	SE
**R G_front_sup**	3.716	NVLD	16	0.043	0.172	0.043
		TD	16	0.281	0.326	0.082
**L S_front_middle**	17.602	NVLD	16	0.017	0.068	0.017
		TD	16	0.295	0.322	0.080

**Table A4:** Bayesian independent samples *t*-test (BF = Bayes factor) and group descriptive (mean, SD = standard deviation, and SE = standard error) of the nonverbal learning disability (NVLD) and the control groups (TD). The dependent variable is the *clustering coefficient* in the *ventral attention network* in both beta and gamma frequency bands.

Beta Frequency Band	BF₁₀	Group	*n*	Mean	SD	SE
**L G_front_inf-Opercular**	4.386	NVLD	16	0.010	0.039	0.010
		TD	16	0.213	0.302	0.075
**Gamma frequency band**	**BF₁₀**	**Group**	***n***	**Mean**	**SD**	**SE**
**R G_temp_sup-G_T_transv**	7.256	NVLD	16	0.330	0.300	0.075
		TD	16	0.069	0.189	0.047
**R G_temp_sup-Lateral**	11.286	NVLD	16	0.347	0.296	0.074
		TD	16	0.069	0.189	0.047
